# Supplementary material for: Phenotypic plasticity shapes carry-over effects in sea rock-pool mosquitoes
Source: Biol Open. 2026 Mar 24;15(3):bio062033. doi: 10.1242/bio.062033 (PMC13054930; doi:10.1242/bio.062033)
Supplement: Supplementary information [file biolopen-15-062033-s1.pdf]

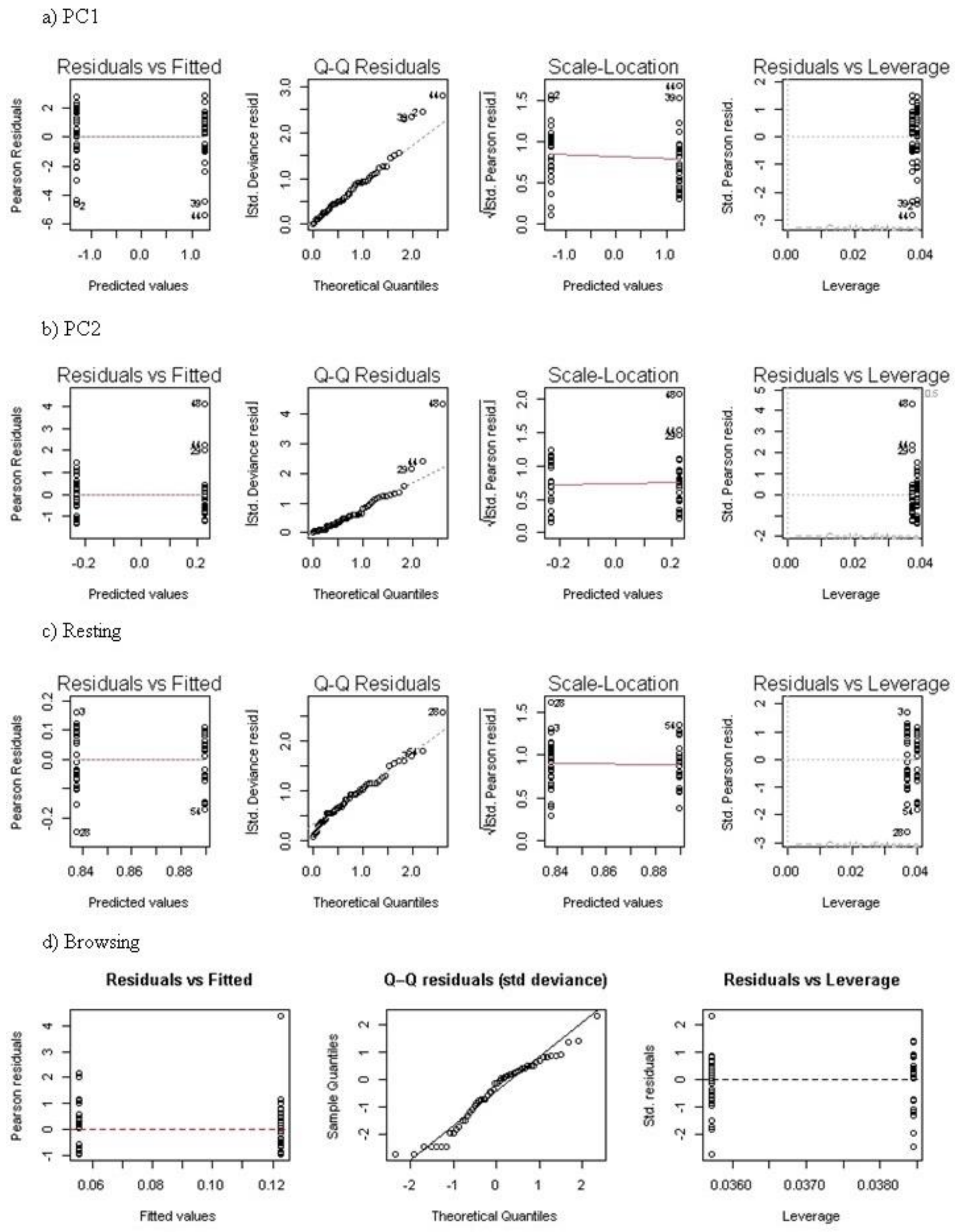

Fig. S1. Diagnostic plots for the fitted models, including residuals versus fitted values, quantile–quantile plots of standardised residuals, scale–location plots and residuals versus leverage. The plots indicate that the Gaussian models (a-c) do not show substantial deviations from normality or heteroscedasticity, while the Gamma model (d) residuals are consistent with the expected variance–mean relationship. No evidence of influential outliers was detected across models.

Biology Open • Supplementary information

Table S1. Results of PCA analysis on morphometric measures of fourth instar larvae

	Eigenvalues	PC1	PC2
**4th instar larvae**			
Proportion of total variance explained		0.769	0.139
	Head width	0.318	0.285
	Thorax width	0.423	
	Abdomen width	0.418	-0.134
	Head length	0.153	0.914
	Thorax length	0.412	-0.192
	Abdomen length	0.413	-0.149
	Total length	0.426	

Table S2. Sequential analysis of deviance table for the GLM testing the effect of larval body size, salinity treatment, and their interaction on pupal size. The model was fitted using a Gaussian error distribution and identity link function. The significant interaction indicates that the relationship between larval and pupal size differs across salinity conditions.

	Df	Deviance	Resid. Df	Resid. Dev	F	Pr(>F)
Larval body size	1	0.096	38	0.975	5.617	**0.023**
Treatment	1	0.086	37	0.888	5.087	**0.030**
Larval body size* Treatment	1	0.272	36	0.615	15.964	**<0.001**

Table S3. Results from SEM analysis to infer the effects of morphology and behaviour of the early stage of development (larvae) on subsequent stages.

Response	Predictor	Non-standardised coefficient	Standardise coefficient	P	Response R^2
**Constant salinity**					
Pupae cephalo-thorax width	Larval body size	0.075	0.891	**<0.001**	0.51
Pupae cephalo-thorax width	Proportion of resting	-0.002	-0.01	0.919	
Proportion of resting	Larval body size	-0.013	-0.249	0.213	0.06
**Increasing salinity**					
Pupae cephalo-thorax width	Larval body size	-0.036	-0.496	0.146	0.17
Pupae cephalo-thorax width	Proportion of resting	-0.172	-0.107	0.720	
Proportion of resting	Larval body size	-0.030	-0.670	**<0.001**	0.45

Table S4. Summary of model diagnostics for the generalised linear models. Pseudo-R^2 is calculated as one minus residual deviance over null deviance and should be interpreted as a descriptive index of explained variance. For the multigroup SEM, the model was saturated with zero degrees of freedom; therefore, global fit indices take perfect values by construction and are not informative. Interpretation focused on the estimated coefficients and the differences between groups.

**Model**	**Distrib.**	**Link**	**n param.**	**LogLik**	**AIC**	**Null dev. (df)**	**Resid. dev. (df)**	**Dispersion**	**Pseudo-R^2**
Morphology-PC1	Gaussian	identity	2	-110.28	224.56	280.18 (52)	191.76 (51)	3.76	0.315
Morphology-PC2	Gaussian	identity	2	-73.62	151.23	50.88 (52)	48.06 (51)	0.94	0.055
Resting behaviour	Gaussian	identity	2	29.33	-54.66	1.11 (53)	1.03 (52)	0.020	0.073
Browsing behaviour	Gamma	log (default)	2	80.30	-156.59	97.09 (53)	88.87 (52)	1.03	0.085
	**Type**	**LogLik**	**AIC**	**BIC**	**χ^2(df)**	**CFI**	**TLI**	**RMSEA (90%CI)**	**SRMR**
SEM	Maximum likelihood	-15.04	58.08	81.37	0 (0)	1.000	1.000	0.000 (0–0)	0.000

Biology Open • Supplementary information

Table S5. Likelihood ratio test (type III) on GLMs for all phenotypic traits with treatment, sex and their interaction as fixed factors.

**Phenotypic trait**	**Fixed factors**	χ^2	**df**	***p*-value**
Morphology- PC1	Treatment	25.666	1	**<0.001**
	Sex	0.644	1	0.422
	Treatment*Sex	3.478	1	0.062
Morphology - PC2	Treatment	2.743	1	0.097
	Sex	0.682	1	0.408
	Treatment*Sex	0.600	1	0.438
Spontaneous activity-Resting behaviour	Treatment	3.900	1	**0.048**
	Sex	3.135	1	0.076
	Treatment*Sex	2.205	1	0.137
Spontaneous activity-Browsing behaviour	Treatment	7.139	1	**0.007**
	Sex	3.781	1	0.051
	Treatment*Sex	3.068	1	0.079
